# QS-21: A Potent Vaccine Adjuvant

**DOI:** 10.4172/2329-6836.1000e113

**Published:** 2015-07-31

**Authors:** Daming Zhu, Wenbin Tuo

**Affiliations:** 1 Laboratory of Malaria Immunology and Vaccinology, National Institute of Allergy and Infectious Disease, National Institutes of Health, 5640 Fishers Lane, Rockville, MD 20852, USA; 2 Animal Parasitic Diseases Laboratory, Agricultural Research Service, USDA, Beltsville, MD 20705, USA

QS-21 is one of the active fractions of the bark of Chilean tree, *Quillaja saponaria*, purified using a reverse-phase chromatography (RP-HPLC) [1–3]. QS denotes its source as *Q. saponaria* and the number 21 as the identity of the RP-HPLC peak [2]. QS-21 is an acylated 3, 28-bisdesmodic triterpene glycosides (1,3) or “saponin” with a molecular formula of C_92_O_46_H_148_ and molecular weight of 1990 Da [1]. It is one of the most potent immunological adjuvants that has been widely used [4–8].

The adjuvant effect of saponins was first reported in 1925, when it was shown that the addition of bread crumbs, tapioca, saponin and “starch oil” to antigenic preparations greatly enhanced antibody responses to diphtheria or tetanus [7,9–11]. In 1951, Espinet used a crude commercially available saponin preparation to increase the potency of foot-and-mouth disease vaccines [10,12]. Further in 1974, Dalsgaard successfully isolated saponin Quil A from the cortex of the South-American Tree *Quillaja saponaria Molina* [3,13–15] and found that Quil A stimulated both humoral and cellular immunity, as well as induced differential antibody isotypes [3, 6,15–17]. Since then, the Quil A has been commercialized and has gained widespread use in veterinary vaccines and pre-clinical studies [3,6,10,14,17–20]. Additional studies showed its effects when co-formulated with aluminum salts, liposomes and oil-in-water emulsions, and with amphipathic proteins and lipids forming detergent/lipid/saponin complexs termed immune-stimulating complexes (ISCOMs) [3,20–23]. However, Quil A is still a heterogeneous product, consisting of up to 23 different saponin peaks detectable by HPLC and its toxicity precluded its use in human vaccines [2–3,6,15,24].

Due to the fact that Quil A is a mixture, a further study was performed by Kensil et al. in 1991[2,6], in which 10 of RP-HPLC fractions from aqueous extract of *Q. saponaria* bark treated by ultrafiltration were tested and found the fractions QS-7, QS-17, QS-18 and QS-21 to be particularly potent. However, their toxicity varies considerably. QS-18, the major component of *Q saponaria*, was found to be highly toxic in mice, while QS-7 and QS- 21 shows far less toxicity [2,6,25]. QS-21 was further extensive studied because it is more abundant than QS-7 [7].

Studies showed that QS-21 promoted high antigen-specific antibody responses and CD8^+^ T-cell response in mice [2–6,17,26] and favored a balanced production of both IgG1 and IgG2a [1,2,27]; in contrast, aluminum hydroxide mainly promotes IgG1 production [2,6,27]. Further studies showed that QS-21 stimulated the production of cytotoxic T-lymphocytes (CTLs), induces Th1cytokines, interleukin-2 and interferon-gamma (IL-2 and IFN-γ), and antibodies of the IgG2a isotype to protein antigens [1,6,26,28,29]. A number of comparative studies has demonstrated that QS-21 significantly outperformed the other classes of adjuvants including glucan formulations, peptidoglycans, amphophilic block copolymers, bacterial nucleosides and bacterial lipopolysaccharides, in augmenting antibody responses as well as T-cell responses against target antigens [2,7].

Due to the high potency, QS-21 has been used as an effective adjuvant with a recombinant retroviral subunit vaccine against feline leukemia virus (FeLV), which is commercially available now [3,6,30].

However, the toxicity and the undesirable haemolytic effect still remain for human use [2,6,7]. QS-21 caused 50% haemolysis of sheep red blood cells (SRBC) at concentrations as low as 7–9 µg/ml (2, 29), and apparently, the side effects associated with QS-21 limit doses to about 50 µg with exception of cancer patient (melanoma, breast and prostate) [1,7]. It is clear that if the higher doses of this adjuvant could be safely used, its immunogenicity should be further increased [2,7]. The Adjuvant System (AS) developed by GlaxoSmithKline demonstrated that the apparent adverse events of QS-21 in rat skeletal muscle could be significantly reduced if QS-21 was formulated in AS01 (composed of liposomes, MPL and QS-21) at a dose of 5 µg, when compared to formulations containing QS-21 alone at the same dose, suggesting that the toxicity of QS-21 can be reduced when formulated with other appropriate adjuvants [5].

QS-21 containing adjuvants such as AS01, AS02 are currently tested in human clinical trials for various vaccine candidates and infectious agents, including influenza, malaria, hepatitis B virus (HBV), human papillomavirus (HPV), HIV/AIDS, tuberculosis (TB), non-small-cell lung carcinoma (NSCLC) and melanoma [1,4,5,8,17,28].

Over the past two decades, the saponin adjuvants have emerged as one of the leading adjuvant candidates. QS-21 containing complex adjuvants in particular have been widely popular in its application to develop vaccines platforms [4,5,7]. It is promising that these QS-21 containing complex adjuvants may be one of the first to be approved as alternative adjuvants in human use in the US in the future.
